# Nanostructured titania films sensitized by quantum dot chalcogenides

**DOI:** 10.1186/1556-276X-6-266

**Published:** 2011-03-29

**Authors:** Athanassios G Kontos, Vlassis Likodimos, Eleni Vassalou, Ioanna Kapogianni, Yannis S Raptis, Costas Raptis, Polycarpos Falaras

**Affiliations:** 1 Institute of Physical Chemistry, NCSR "Demokritos", Aghia Paraskevi Attikis, Athens 15310, Greece; 2 Physics Department, School of Applied Mathematical and Physical Sciences, National Technical University of Athens, Zografou, Athens 15780, Greece

## Abstract

The optical and structural properties of cadmium and lead sulfide nanocrystals deposited on mesoporous TiO_2 _substrates via the successive ionic layer adsorption and reaction method were comparatively investigated by reflectance, transmittance, micro-Raman and photoluminescence measurements. Enhanced interfacial electron transfer is evidenced upon direct growth of both CdS and PbS on TiO_2 _through the marked quenching of their excitonic emission. The optical absorbance of CdS/TiO_2 _can be tuned over a narrow spectral range. On the other side PbS/TiO_2 _exhibits a remarkable band gap tunability extending from the visible to the near infrared range, due to the distinct quantum size effects of PbS quantum dots. However, PbS/TiO_2 _suffers from severe degradation upon air exposure. Degradation effects are much less pronounced for CdS/TiO_2 _that is appreciably more stable, though it degrades readily upon visible light illumination.

## Introduction

In recent years, nanostructured materials and quantum dots (QDs) light harvesting assemblies have emerged as highly promising building blocks for the development of and third generation solar cells affording efficient conversion of solar energy to electricity. Among different technologies, dye sensitized solar cells (DSCs) [1] hold great promise as an alternative renewable energy system with the advantages of low cost, transparency and flexibility [2]. DSCs make use of nanocrystalline semiconducting electrodes (the most common being TiO_2_) sensitized with molecular dyes (the most efficient being polypyridyl ruthenium(II) complexes) in order to harvest solar light. In contrast to conventional *p*-*n *type devices, charge separation in DSCs takes place at the photoelectrode/sensitizer interface via electron injection from the dye into the conduction band of the semiconductor, followed by diffusive electron transport through the interpenetrated mesoporous network of the TiO_2 _semiconductor to the charge collector, while dye regeneration occurs via a redox electrolyte. Even though such devices have reached high performance and stability standards [3], the prospect of developing inorganic hybrid heterojunctions with enhanced selectivity, efficiency and robustness offering cost reduction and simplification in the DSCs manufacturing is attracting a great deal of attention.

One of the most attractive approaches for the utilization of inorganic heterojunctions in DSCs is the exploitation of the exceptional electronic properties of chalcogenide such as CdS, CdSe, PbSe, PbS and CdTe nanocrystals as light harvesting antennas [4-6]. Based on the unique quantum confinement effects, QDs offer unique high extinction coefficients and band gap tunability from the visible to the infrared spectral range by size control. Moreover, they can form favourable QDs/TiO_2 _as well as QDs/dye/TiO_2 _heterojunctions for efficient charge extraction [7-11]. A major drawback underlying the relatively low light harvesting ability and the concomitant reduced photocurrents in quantum dot sensitized solar cell devices is the amount of QDs adsorbed on the TiO_2 _electrode. Two main approaches have been so far exploited for the sensitization by QDs: *in situ *growth of QDs on TiO_2 _by chemical bath deposition (CBD) [7,12] and successive ionic layer adsorption and reaction (SILAR) [13,14] or attachment of preformed colloidal QDs to the TiO_2 _mesoporous structure by means of bifunctional linker molecules or direct adsorption using a suitable solvent in the colloidal solution [8,11]. Linker-assisted and direct QD adsorption onto TiO_2 _allows fine control of the QD size, exploiting colloidal synthesis. However these systems suffer from rather low QD loading and relatively weaker electronic coupling between QDs and TiO_2_. On the other hand, CBD permits enhanced electron transfer to the wide band gap TiO_2 _electrode and significantly higher loading at the cost of appreciable QD aggregation that finally deteriorates solar cell performance [5,6]. On the contrary, direct growth of QDs by SILAR has recently emerged as a promising deposition route combining high QD loading together with low degree of aggregation and efficient electron transfer to TiO_2 _[14,15].

In this work, we report a comparative investigation on the direct growth of chalcogenide CdS and PbS nanocrystals spanning a wide spectral range for light absorption on mesoporous TiO_2 _films employing the SILAR method. Reflectance and transmittance together with micro-Raman measurements were exploited to identify the optical and structural properties as well as quantum size effects of the sulfide nanocrystals and their stability upon air and light exposure. The electron injection efficiency of the sensitized films was accessed by photoluminescence (PL) measurements and the variation of the QD emission signal upon grafting onto TiO_2_.

## Experimental

Mesoscopic TiO_2 _films of a thickness of 15 μm were prepared using a TiO_2 _paste made of Degussa P25 nanoparticles on glass substrates, followed by sintering at 450°C [16]. Films present excellent adherence to the glass substrate. For the CdS SILAR deposition [14], the TiO_2 _films were pretreated with a quick soaking in 1 M NH_4_F aqueous solution. Then, they were dipped into 0.05 M Cd(NO_3_)_2_, ethanol solution, rinsed in pure ethanol to remove excess of the precursor and dried in air. The same process was followed for depositing S^2-^, by successive dipping the films in 0.05 M Na_2_S solution, rinsing in pure methanol and drying. Each individual step lasted for 1 min and a total of 9 SILAR cycles were employed. PbS deposition was likewise carried out by sequential immersing the TiO_2 _film initially in a 0.02-M Pb(NO_3_)_2 _methanol solution, and then to a 0.02-M Na_2_S methanol solution. The process starts and terminates with Pb^2+ ^deposition accomplishing 5.5 SILAR cycles [14].

Diffuse reflectance (*R*) and transmittance (*T*) measurements were carried out employing a Hitachi 3010 spectrophotometer equipped with a 60-mm diameter integrating sphere. The absorbance (*A*) spectra were derived as *A *= 1 - *R *- *T*. Surface morphology was examined with a digital Instruments Nanoscope III atomic force microscope (AFM), operating in the tapping mode. Micro-Raman and PL measurements were performed at room temperature employing a vacuum cell equipped with an optical window. For Raman, a Renishaw inVia spectrometer was employed, using an Ar^+ ^ion laser (λ = 514.5 nm) and a high power near infrared (NIR) diode laser (λ = 785 nm) as excitation sources for CdS and PbS QDs, correspondingly. The spectra were recorded by focusing the laser beam on the film surface and controlling the light power to give 0.01 to 0.2 mW/μm^2 ^at about 1.5 μm diameter spot. For PL experiments in PbS, the above facility was used, while for CdS, excitation of the film was done by focusing the 476.5-nm line of an Ar^+ ^laser at 20 mW on the sample surface with an 8-cm focal length cylindrical lens. The emitted radiation was analyzed through a SPEX double monochromator, followed by photomultiplier detection.

## Results and discussion

**Figure 1:**
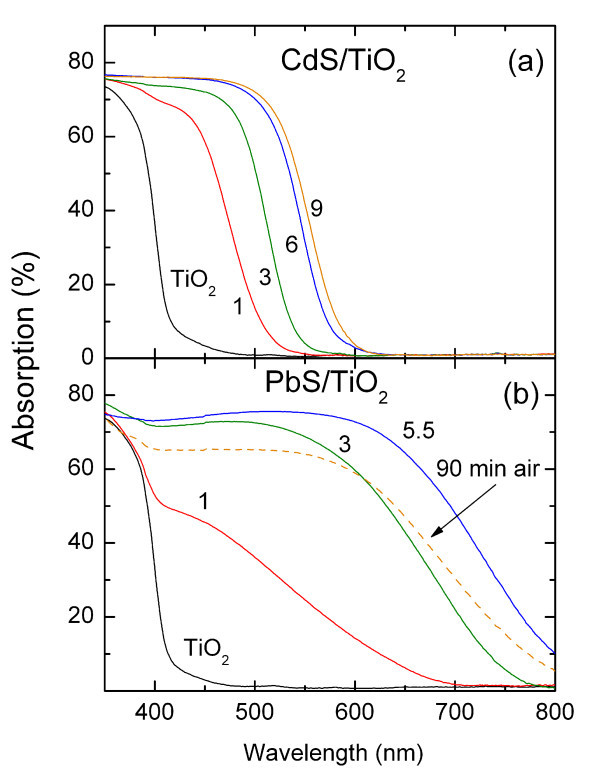
**Absorbance spectra of the mesoporous TiO_2 _films upon SILAR deposition of (a) CdS and (b) PbS**. Numbers correspond to the different SILAR cycles. The spectra of PbS/TiO_2 _after 90 min air exposure are also included in **(b)**.

Figure 1a shows the evolution of the CdS/TiO_2 _absorbance, calculated from the corresponding transmittance and reflectance spectra, for successive SILAR cycles compared to that of the bare TiO_2 _films. Significant absorption in the visible range is thus observed, indicating the formation of CdS nanocrystals with gradually increasing concentration with the SILAR cycles. However, the distinct excitonic peaks, commonly observed for colloidal CdS QDs with a narrow size distribution, cannot be resolved, implying rather broad size dispersion for the SILAR deposited QDs. Moreover, the CdS/TiO_2 _absorption edge reached 585 nm upon completion of the ninth coating cycle. This value is close to that expected for bulk CdS, whose energy gap is approximately 2.4 eV, complying with the formation of nanocrystals with size exceeding 6 nm, above which quantum size effects essentially cease for CdS QDs [17]. On the other hand, an appreciable increase of the mean CdS particle size can be inferred from the gradual red-shift of the absorption edge, most prominent for the initial SILAR cycles. This is indicative of weak quantum size effects, pertaining for CdS nanocrystals with diameters slightly below 6 nm.

Figure 1b shows the corresponding evolution of the PbS/TiO_2 _absorbance spectra with the SILAR cycles. In that case, the absorption edge of the sensitized system extended well in the NIR spectral region, presenting a marked shift from 690 nm for the first SILAR cycle up to 840 nm for the last PbS coating. These wavelengths are much shorter than the absorption edge (approximately 3000 nm) of bulk PbS that possess a narrow band gap of only 0.41 eV. This distinct variation of the PbS/TiO_2 _absorbance reflects essentially the large exciton Bohr radius (approximately 18 nm) of PbS QDs, affording wide tunability through the pronounced quantization effects for PbS nanocrystals over an extended particle size [18]. Even though the broad spectral absorption of PbS/TiO_2 _is expected to comprise appreciable contributions from the whole electronic spectrum of the underlying PbS nanocrystals, its strong dependence on the coating cycles verifies that direct growth of PbS QDs on TiO_2 _and their optical response can be efficiently tuned by the SILAR technique through a broad size/spectral range.

However, storage of the PbS/TiO_2 _films under ambient conditions produced rapid degradation of their optical response. Specifically, brief exposure of the PbS/TiO_2 _to air for 90 min resulted in the drastic decrease of the absorbance and the shift of the absorption edge to shorter wavelengths, indicative of the reduction of the PbS size, as shown by the dashed line in Figure 1b. This variation can be associated with the prominent tendency of lead sulfide towards surface oxidation at ambient conditions, which is especially detrimental for the larger PbS nanocrystals [19]. Storage under vacuum conditions in evacuated cells was accordingly found to be necessary to retain the PbS/TiO_2 _spectral characteristics intact. Similar degradation effects were also observed for the CdS/TiO_2 _films upon air exposure, though much less severe than those on PbS/TiO_2_, indicating their higher resistance to air oxidation that can be largely prevented by storage under inert atmosphere.

QD nanoparticles can be hardly identified in SEM and AFM images of the films, due to the rough characteristics of the TiO_2 _nanostructured substrate film. However, a morphological evidence of the CdS QDs came from 1 × 1 μm AFM surface images (not shown) on nanoparticulate sol-gel anatase TiO_2 _(chosen as a reference substrate) and comparing it with the surface of the CdS/TiO_2 _film corresponding to the full set of the 9 SILAR cycles. Thus, significant enhancement of the surface roughness was observed (Rms = 15.9 nm for CdS/TiO_2 _vs. 6.6 nm for bare TiO_2_), due to the CdS QDs growth on the surface, in agreement with literature [7].

**Figure 2:**
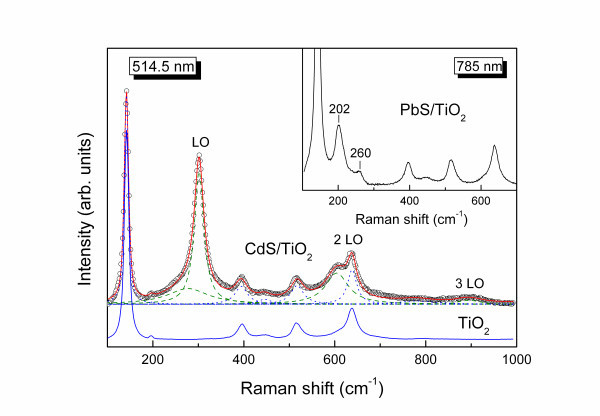
**Resonance Raman spectrum of CdS/TiO_2 _in comparison with the bare TiO_2 _film, at 514.5 nm**. *Dashed *and *dotted lines *depict the spectral deconvolution to the CdS and TiO_2 _vibrational modes, respectively. The *inset *shows the Raman spectrum of PbS/TiO_2 _at 785 nm.

The structural characteristics of the QD sensitized TiO_2 _films were investigated by resonance Raman measurements under vacuum in order to avoid air degradation. Figure 2 shows the Raman spectrum of CdS/TiO_2 _(9 SILAR cycles) at 514.5 nm, which is close to the absorption edge of the CdS nanocrystals and thus allows their resonant excitation. The characteristic Raman-active phonons of the underlying TiO_2 _substrate can be readily identified in comparison with the bare TiO_2 _electrode, the most intense being the low frequency anatase *E*_g _mode at approximately 142 cm^-1 ^[3], together with the resonantly excited longitudinal optical (LO) phonon of CdS QDs at approximately 300 cm^-1 ^[20]. Spectral analysis reveals a slight asymmetric broadening of the CdS LO mode at the low frequency side, which can be effectively fitted to the superposition of two peaks, the LO mode at 301 cm^-1 ^with full width at half maximum (FWHM) of 25 cm^-1 ^and a broad low frequency mode at 277 cm^-1 ^with FWHM of approximately 109 cm^-1^. Moreover, resonant excitation allows identifying the first (2 LO) and second (3 LO) overtones of the CdS nanoctystals at 604 and approximately 900 cm^-1^, respectively. The frequency of the LO peak matches bulk CdS (301 cm^-1^), whereas its width is considerably larger than the corresponding bulk value (approximately 12 cm^-1^) [20]. The broadening of the LO peak together with its asymmetric lineshape corroborates the presence of a broad size distribution of CdS nanocrystals and the absence of strong phonon confinement effects [21], in agreement with the features of the CdS/TiO_2 _optical absorbance.

Raman measurements under NIR excitation (785 nm) were applied to identify the structural integrity of the lead sulfide nanocrystals through resonance excitation on the PbS/TiO_2 _films. A composite band comprising two bands at 202 and 260 cm^-1 ^could be accordingly resolved on the sensitized PbS/TiO_2_, as shown in the inset of Figure 2. Lead sulfide crystallizes in rock salt structure precluding first-order Raman scattering from phonons near the centre of the Brillouin zone (*k *= 0). However, the formally 'forbidden' LO scattering at 200 to 215 cm^-1 ^may become allowed under conditions of resonant or quasi-resonant Raman excitation via the Fröhlich interaction, while appreciable contributions may also arise at these frequencies from two-phonon scattering of longitudinal acoustic and transverse optical modes in PbS [22]. A characteristic broad Raman band has been also reported at approximately 430 cm^-1 ^due to 2 LO scattering in PbS [22], which, however, cannot be safely discriminated in the PbS/TiO_2 _spectra due to the additional contribution of the rutile TiO_2 _phonon at approximately 447 cm^-1^.

**Figure 3:**
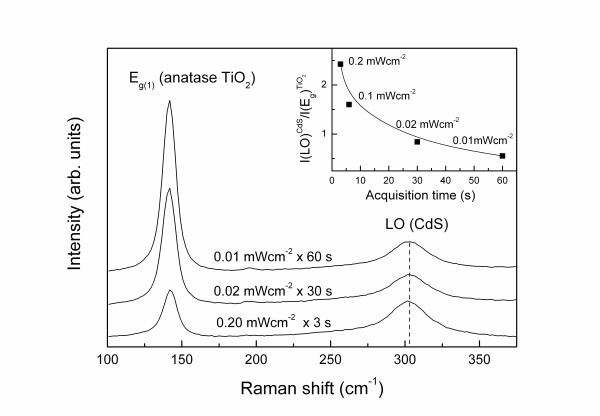
**Evolution of the CdS/TiO_2 _Raman spectra upon simultaneous variation of the laser power and acquisition time (irradiation dose remains constant)**. The *inset *shows the variation of the intensity ratio *I*(LO)^CdS^/*I*(*E*_g_)^TiO2 ^determined from the integrated areas of the CdS LO mode and the *E*_g _anatase TiO_2 _mode, with the spectral acquisition time.

Degradation effects were also observed in the CdS Raman signal when acquired in ambient conditions, though considerably less pronounced than those of PbS/TiO_2_. Most importantly, an intriguing photodegradation effect on the CdS Raman intensity was evidenced by varying the laser irradiation time in ambient conditions. Figure 3 shows characteristic resonance Raman spectra of CdS/TiO_2 _acquired in air under variable laser power density and different acquisition times so that the total irradiation dose (product of laser power × acquisition time) remains constant. In that case, a marked increase of the CdS LO Raman intensity relative to that of the *E*_g _anatase TiO_2 _mode occurred by decreasing the spectral acquisition time (inset of Figure 3). Ordinary local heating effects are excluded since the relative CdS LO intensity was found to increase with the laser power and no appreciable shift and broadening of the LO mode or variation of the *I*_2LO_/*I*_LO _intensity ratio were identified [20], indicating that the observed behavior is related to the duration of exposure of the CdS/TiO_2 _films to the laser beam. This variation was completely suppressed when Raman experiments were conducted in an isolated cell compartment under vacuum conditions, pointing to a photodegradation effect of the CdS nanocrystals under ambient conditions. A similar result was recently reported for CdSe QDs anchored to TiO_2 _following visible light irradiation under atmospheric conditions [23]. In that case, time resolved transient absorbance and emission measurements revealed that electrons injected from CdSe to TiO_2 _may be scavenged by surface adsorbed oxygen leaving behind reactive holes, which cause anodic corrosion of the CdSe QDs. An analogous mechanism can be accordingly proposed for the CdS/TiO_2 _system upon resonant laser irradiation at 514.5 nm, causing electron injection to TiO_2 _and the surface oxidation of CdS nanocrystals through the remaining valence band holes.

**Figure 4:**
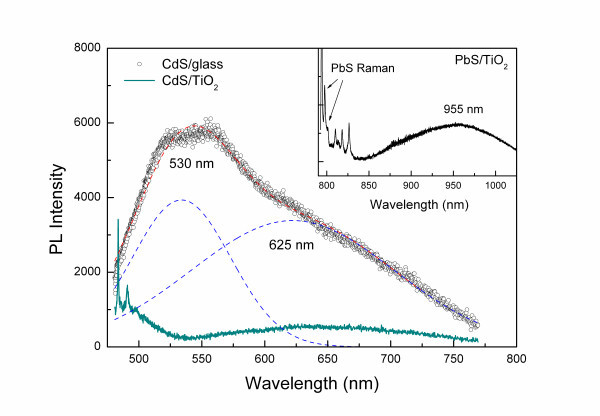
**PL spectra of CdS/glass and CdS/TiO_2 _films excited at 476.5 nm**. The *inset *shows the PL spectrum of PbS/TiO_2 _obtained with 785 nm excitation.

Figure 4 shows the PL spectra acquired simultaneously with the Raman signal of the CdS/TiO_2 _under anaerobic conditions. To explore the charge injection efficiency for the QDs to the TiO_2 _substrate, CdS nanocrystals were deposited on microscopic glass employing 9 SILAR cycles, leading to a film with similar optical and Raman spectroscopic characteristics to that grown on TiO_2_. Comparison of the corresponding PL spectra, after subtraction of the relatively weak emission of the glass substrate, reveals significant changes between the CdS/TiO_2 _and CdS/glass films. The PL spectra of CdS/glass exhibits a strong component at about 530 nm, which is close to the band gap emission of bulk CdS arising from radiative excitonic recombination, while a rather broad emission band occurs at 625 nm most likely due to the recombination of trapped carriers by defect states [24]. The frequency of the former emission band indicates the absence of significant quantum size effects, further supporting the growth of nanocrystals with size appreciably larger than the Bohr exciton radius of CdS (approximately 2.8 nm). Moreover, the width of the CdS excitonic peak (FWHM ~ 80 nm) in the CdS/glass film exceeds largely that of bulk CdS (FWHM ~ 20 nm) [24], indicative of a broad size distribution of the SILAR deposited CdS nanocrystals. However, upon CdS deposition on TiO_2_, the PL intensity of the excitonic emission is drastically suppressed, verifying the effective quenching of the radiative recombination of photoexcited carriers by electron transfer from CdS to TiO_2_.

In the case of PbS/TiO_2_, the PL emission spectra could be detected simultaneously with the Raman signal at 785 nm excitation. A very weak and broad PL band could be thus traced at 955 nm after subtraction of the glass background, as shown in the inset of Figure 4. This emission band emerges at wavelengths just above the absorption edge of the PbS/TiO_2 _(approximately 840 nm), complying with the excitonic PL of an ensemble of PbS QDs with a broad size distribution around 3 nm [25]. Moreover, the PL emission band could be resolved only for freshly sensitized films PbS/TiO_2_, while it degraded rapidly upon air exposure verifying the great sensitivity of the system to surface oxidation. The drastic reduction of excitonic emission evidenced for both CdS and PbS nanocrystals upon direct growth on TiO_2 _by SILAR, markedly weaker than the emission colloidal QDs adsorbed on TiO_2 _[11,23], verifies the great potential of this deposition technique to enhance electronic coupling and the concomitant charge transfer between QDs and the underlying TiO_2 _substrate.

## Conclusions

CdS and PbS nanocrystals can be efficiently deposited as sensitizers on mesoporous TiO_2 _substrates via the SILAR method. Enhanced electronic coupling and interfacial electron transfer are confirmed upon direct growth of the chalcogenide nanocrystals on TiO_2 _through the marked quenching of their excitonic emission. The optical absorbance of CdS/TiO_2 _can be tuned over a narrow spectral window in the visible range, reflecting essentially the small exciton Bohr radius of CdS QDs that inhibits utilization of quantum size effects for light harvesting. On the other hand, PbS/TiO_2 _exhibits pronounced band gap tunability spanning the visible to the NIR range, due to the prominent quantum size effects of PbS QDs. However, PbS/TiO_2 _degrades severely upon air exposure requiring a protection layer for application in solar cell devices. In contrast, CdS/TiO_2 _is appreciably more stable under ambient conditions, though it degrades readily under visible light irradiation.

## Abbreviations

AFM: atomic force microscope; CBD: chemical bath deposition; DSCs: dye sensitized solar cells; FWHM: full width at half maximum; NIR: near infrared; PL: photoluminescence; QDs: quantum dots; SILAR: successive ionic layer adsorption and reaction.

## Competing interests

The authors declare that they have no competing interests.

## Authors' contributions

AGK participated in the design and implementation of the work and help to draft the manuscript. VL carried out the Raman characterization and analysis. EV carried out the preparation of CdS QDs on TiO_2_. IK carried out the preparation of PbS QDs on TiO_2_. YSR participated in the realization of the photoluminescence experiments. CR have been involved in revising the manuscript critically for important intellectual content. PF conceived the study, participated in its design and coordination, and helped to draft and finalize the manuscript. All authors read and approved the final manuscript.
